# The Influence of Multiple Mechanical Recycling of Particleboards on Their Selected Mechanical and Physical Properties

**DOI:** 10.3390/ma15238487

**Published:** 2022-11-28

**Authors:** Anita Wronka, Grzegorz Kowaluk

**Affiliations:** Institute of Wood Science and Furniture, Warsaw University of Life Sciences—SGGW, Nowoursynowska St. 159, 02-776 Warsaw, Poland

**Keywords:** particleboard, recycling, upcycling, wood, composite, furniture, raw material, mechanical properties, physical properties, circular economy

## Abstract

This is a bridge between circular economy issues and wood-based panels technology, especially particleboards. Because these composites contain a significant amount of non-wood raw material (10–12% thermoset resin, high hardness laminates, among others), their mechanical recycling leads to an uncontrollable reduction in produced particle size. This problem can be especially significant since the particleboards can be intended for multiple recycling due to the shortening of their service life. This research aimed to produce particles in the cycle of multiple re-milling particleboards and evaluate the selected properties of the produced particles and particleboards. Thus, the response to the following scientific problem can be given: what factors qualitatively and quantitatively influence the properties of the particleboards produced by multi-re-milled particles? The novelty of this research is the approach to recycling the raw materials from particleboards in fully controlled conditions, providing the characterization of produced particles and producing particleboards with close-to-industrial parameters, and, finally, evaluating the features of produced particleboards in the light of raw materials used. The results confirmed that subsequent mechanical recycling of particleboards, where the other panels are made entirely of second-milling particles, leads to an unprofitable and unacceptable reduction in the mechanical properties of the panels. The physical parameters, such as thickness swelling and water absorption, are improved, but this can be the result of increased content of chemical ingredients, which negatively influence the hygienic features of panels (emission of formaldehyde and total volatile organic compounds—TVOC). Further research should be directed towards estimating the optimal addition of mechanically recycled particles to particleboard production.

## 1. Introduction

Wood recycling is not the easiest of tasks, especially when analyzing the recycling of wood composites such as particleboard, plywood, and medium-density fiberboards (MDF). The composites mentioned above often contain formaldehyde in the glue composition, which significantly limits the reuse of raw wood material due to difficult disposal. However, there are promising attempts to develop formaldehyde-free binders for wood composites [1] (for example, bonding the boards with cassava-based starch adhesive [2]). The wood-based composite industry is still concerned about formaldehyde-containing resins. The most common way to dispose of wood waste containing formaldehyde-based binders is energy recovery by combustion under special conditions. Unfortunately, this action contributes to the emission of numerous chemicals such as nitrogen oxides, sulfur oxides, dioxins and polychlorinated biphenyls, and carbon monoxide [3]. Currently, there are frequent shortages of raw materials in the wood market. Their prices have been hitting record highs in the past few years, so research is also constantly being developed on raw materials that can be a substitute for wood in wood plastics technology; such raw materials can include agricultural biomass [4], apple and plum tree branches [5], vine pruning stalks [6], pepper stalks [7], and palm branches [8]. In this case, recycling is perceived to be a better option than disposing of this wood waste in landfills or burning it and thus polluting the environment through volatile compounds [9]. There are various methods of treating wood for reuse, but none are yet used on a broader scale, making wide-scale wood recycling much more complex [10]. One such method is the removal of urea-formaldehyde (UF) adhesives from waste wood particleboard by hydrolysis [11]. One of the advantages of wood substitution is the reduction in greenhouse gas emissions released into the atmosphere [12]. The keyword analysis is unambiguous, and a significant increase in interest in wood recovery topics has been noted over the last decade [13].

Unfortunately, due to the nature of the waste, post-use wood recycling involves mostly small-sized products, such as particles, fibers, and dust [14]. This phenomenon is called downcycling, and it is ubiquitous in the woodworking industry, where wood is recycled to make new products, resulting in a new product of lower value (utility or technical) [15].

Wood recycling encounters many problems along the way in terms of species, storage method, and types of contamination—some will be dry, some wet, and some pieces of wood will have nail residue. Research confirms that too high a moisture content of the particles used may adversely affect the production process, leading to delamination of the particleboard during pressing [16]. On the other hand, maintaining the appropriate values of moisture content or raising it in the case of excessively dry wood allows for the optimization of production parameters, thus reducing costs [17], in addition, raising the outlet in the face layers allows for better heat transfer during ironing to the core layer [18]. Therefore, there has been much discussion on the appropriate way to manage wood waste so that wood is valuable for as long as possible and has a high economic value [19]. Managing waste processing is only part of the overall process of dealing with the problem. An additional aspect remains the logistics of waste collection and proper waste selection, which would improve processing. To date, there have been attempts to select various wood wastes in the library using 3D scanning, which has been used to design buildings [20]. These issues should be considered at the design stage of a given product, based on the recovery of the raw material used in the future. The subsequent step is consumption. If working on a leasing basis, the customer could return, for example, a furniture item to the manufacturer or an intermediate point at a suitable time. Combining all of these components with logistics and again with the manufacturer gives greater control over the preservation of the quality of the raw material because the higher it is, the greater the chances of reuse in this case of wood [21]. These issues were also mentioned in the research of Nautiyal et al., where it was also aptly stated that a large portion of the wood that could still be a material for producing wood composites is fuel material in factories [22]. Another example of the use of wood by-products from a sawmill is the production of sawdust particleboard [23].

Nonetheless, there is great potential for improving recycling and upcycling processes in the wood sector to meet future EU requirements related to reducing carbon emissions planned for the coming years [24]. Wood recycling in the form of cascade wood utilization provides a new perspective on using post-use wood [25]. It contributes to saving raw materials and thus binds carbon monoxide for longer in the atmosphere, slowing climate warming [26].

In the case of boards for furniture, it is worth mentioning that they comprise not only wood and binders but also very often various varnishes or finishes. The simplest solution seems to be the removal of existing finishes, but this process increases costs and requires special technological facilities. The research has now confirmed the possibility of using dried laminate for board lamination. The present laminate was shredded and then added to particleboards. The results confirmed the increased strength of the particleboards produced and suggested that using shredded laminate makes it possible to reduce urea-formaldehyde adhesive usage (UF) [27]. Similar studies that have been carried out also confirm the possibility of using laminate as a binder substitute. In addition, it was determined that the size of the laminate particles affects the sealing quality [28]. The strength parameters of the manufactured wood materials can also be improved by manipulating the amount of laminate and glue used in the individual layers of the three-layer particleboard [29]. The present study shows that it is likely that the removal of the surface as laminates will not be necessary, and sometimes it may even contribute to improving the performance of the manufactured panels or bonding them. Increased laminate content in the composition of particleboard can contribute to faster dulling of the saw blade during processing. The hard particles of laminate can significantly influence the precision of mechanical processing of the materials containing these reused particles [30]. Thus, the adhesive content also increases, which can lead to increased formaldehyde emissions. A way to reduce formaldehyde emissions is to use suitable compounds, such as urea, ammonia, ammonium salts, and some natural compounds, such as tannin and wood bark [31].

One example of recycling post-consumer wood composites is plywood recycling. In this case, plywood waste from two manufacturing plants in Poland was used. The plywood used was bonded with UF and phenol-formaldehyde (PF) resin. It was waste from edge processing. A wood shredder with screens of 10, 14, 25, and 38 mm mesh was used to shred the present waste. The particles obtained produced three-layer particleboards with a thickness of 16 mm and a density of 650 kg m^−3^. The study proved that the nature of the binder used in the plywood impacts the performance of the particleboard produced; in this case, particles from plywood glued with UF adhesive performed better [32]. An additional obstacle in this process is that the wood used in plywood becomes denser, reducing its porosity, which translates into poorer penetration and wetting of the surface [33]. Another difficulty that can be found on the path of raw material recycling is the geometry of the resulting particles. Depending on the method of shredding wood composites and the degree of gluing, the recovered raw material is shorter, and its shape is no longer as sleek as in the case of commercial particles [34,35]. In addition, the proportion of the dusty part increases. Based on the study, it was noted that a wood particle in the particleboard for more than a few cycles (5–10) degrades significantly to dust, which is usually burned at the final stage since this waste is the most sensitive to management [36]. Other methods used to extend the service life of plywood are the addition of carbon fiber and glass fiber in its production, which increases strength parameters [37,38].

Another type of wood that can be recycled in this case to make particleboard is wood from construction and demolition waste. The advantage of this solution is that by using demolition wood, we somewhat reduce the drying process of virgin wood, thus saving energy [39]. The raw material was obtained from a company that recycles wood, so the authors relied on four materials: MDF, particleboard, plywood, and wood. The wastes, as mentioned earlier, were shredded into appropriate fractions, and then particleboard with a density of 750 kg m^−3^ was made from them, and urea-formaldehyde glue was used for bonding. Based on the research, it was determined that the recovered raw material could be used as filler in the core layer of the particleboard, but the process itself needs to be improved due to the lower strength parameters of modulus of rupture (MOR) and modulus of elasticity (MOE) in static bending [40]. Americans have high hopes for the deconstruction of timber-framed houses. It is believed that this approach is much more efficient than demolishing buildings; thus, there is a chance to recoup some of the costs incurred. In addition, deconstruction allows for the recovery of low-cost, high-quality wood materials and significantly reduces the carbon footprint [41]. Demolition waste and wood from pallets are used, among other things, for wood wool cement composite, which is used in the construction industry [42].

Another way to recover wood from derived particleboard is through hydrothermal treatment. Four different hydrothermal (steam) treatments were used to recover the wood under different pressure–temperature–length conditions (2 bar/119 °C/480 min; 4 bar/140 °C/120 min; 6 bar/156 °C/45 min; 8 bar/167 °C/20 min). In addition, the commercial particleboard produced was not shredded or treated with any solutions to aid in its degradation. The process of recovering the raw wood material made the wood particles smaller, thus increasing the bulk density of the raw material. After the tests, a decrease in internal bonding in the boards and strength at axial screw pullout and MOR were found. Improvements were noted for MOE, where this parameter may have been affected by hydrothermal treatment of the wood [43].

The research conducted to date in furniture processing and its use in particleboard production has consisted of milling furniture particleboards and adding them to commercial particles. Particleboards in proportions of 10, 25, 50, 75, and 100% have been produced. The particleboards produced had a density of 700 kg m^−3^, and urea-formaldehyde resin was used to seal them in a proportion of 10% solids—the dry weight of particles and 1% paraffin emulsion. The study showed the potential of using milled furniture particleboards to produce new particleboard [44].

This literature review confirms that few studies are focusing on reprocessing wood composites, which may indicate the potential of this topic. Additionally, solutions that will have the potential for industrial application are still being explored. However, even if particleboards made of recycled particleboard wastes are commercially available [45], there is no clear information about the possibilities and limitations of multiple recycling of wood-based composites, for example, particleboards.

**Figure 1:**
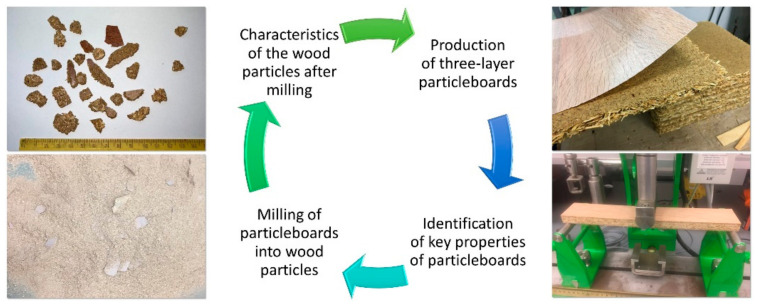
The general principle of the research.

This research aimed to produce the particles by multiple re-milling particleboards to fill this gap and evaluate the selected properties of the produced particles and particleboards. The general principle of the research is presented in Figure 1. As shown, there are several attempts to investigate the properties of wood-based composites made of recycled materials. However, this approach aims mainly at the single recycling of raw materials. Given the challenges of the circular economy, the single recycling of raw materials, and the knowledge of the circumstances of recycling and its influence on the produced goods, the complete information required to fully switch from a linear economy and implement a circular economy (closing the loop) in particleboard production is not yet available. The novelty of this research is the approach to recycling the raw materials from particleboards in fully controlled conditions, providing the characterization of produced particles and producing the particleboards with close-to-industrial parameters, and, finally, evaluating the features of produced particleboards in the light of raw materials used. Thus, the response to the following scientific problem can be given: what factors qualitatively and quantitatively influence the properties of the particleboards produced by multi-re-milled particles?

## 2. Materials and Methods

### 2.1. Materials

Industrial particles, pine *Pinus sylvestris* L., about 3% moisture content (MC), intended for use in face and core layer particleboard production and received from a plant in Poland, were used. As a binder, an industrial urea-formaldehyde (UF) resin Silekol S-123 (Silekol Sp. z o. o., Kędzierzyn—Koźle, Poland) of about 66% dry content [46] was used, with ammonium nitrate water solution as a hardener, to reach the curing time of gluing mass in 100 °C of about 86 s. The addition of 1% (dry content) of industrial paraffin emulsion, received from a plant in Poland, was applied for face and core layer particles during blending.

### 2.2. Preparation of Panels

An air gun was used to spray the glue over the particles during blending in a laboratory blender. The nominal density of the panels was 680 kg m^−3^, and the mass share of the face layers was 32%. The manually formed mats were initially pre-pressed on a hydraulic press (ZUP-NYSA PH-1P125) at room temperature for 30 s under the maximum unit pressure of 0.9 MPa and then pressed in a hot (180 °C) hydraulic press (AKE, Mariannelund, Sweden) with a press factor of 20 s mm^−1^ of the nominal thickness of the panel under the maximum unit pressure of 2.5 MPa. The maximum press pressure was kept for the initial 50% of the pressing time, then was reduced by 1/3 for the next 20% of the pressing time, then again reduced by 1/3 for the next 20% of the pressing time, and after this, the pressure was continuously reduced to open the press within the remaining 10% of the total pressing time. The produced boards were conditioned before the tests at 20 °C and 65% ambient air humidity until a constant mass had been obtained.

After conditioning, the face layers of the panels were covered by industrial melamine-impregnated decorative paper delivered by a particleboard plant in Poland. The following parameters have been applied to bond the melamine paper to panel surfaces: top shelf temperature 200 °C, bottom shelf temperature 190 °C, pressing time 40 s, unit pressure 0.65 MPa.

### 2.3. Characterization of the Elaborated Panels

The test specimens were cut on a saw blade as required by European standards EN-326-2 [47] and EN-326-1 [48]. The modulus of rupture (MOR) and modulus of elasticity (MOE) were determined according to EN-310 [49], the internal bonding (IB) was determined according to EN-319 [50], and the screw withdrawal resistance (SWR) was measured according to [51]. All the mechanical properties were examined with a computer-controlled universal testing machine delivered by the Research and Development Centre for Wood-Based Panels Sp. z o. o. (Czarna Woda, Poland). The selected results, whenever applicable, were determined in accordance with European standards [52]. Board density was determined according to EN-323 [53] and thickness swelling (TS) and water absorption (WA) according to EN-317 [54]. The density profiles of the tested panels were measured on a GreCon DAX 5000 device (Fagus-GreCon Greten GmbH and Co. KG, Alfeld/Hannover, Germany). The formaldehyde (HCHO) and total volatile organic compounds (TVOC) emission was determined according to [55] on two samples per tested panel. The number of samples of every tested panel was less than 10 (3 for the density profile) for every abovementioned test. The density profile measurement results are the representative plots selected after analyses of 3 individual plots for every tested panel. The average and standard deviation values of achieved results are shown in Table 1.

### 2.4. Recovering and Size Characterization of the Raw Material

The samples of the produced panels after the mechanical tests were re-milled on a laboratory knife mill (laboratory prototype provided by Research and Development Centre for Wood-Based Panels Sp. z o. o. in Czarna Woda, Poland) equipped with knives and contra-knifes as found in dedicated industrial particleboard production cutters. The fractions of particles (fresh and taken from the re-milled particleboards) were tested with an IMAL (Imal s.r.l., San Damaso (MO), Italy) vibrating laboratory sorter with seven sieves. The selected sieve sizes were 8, 4, 2, 1, 0.5, and 0.25 mm. The amount of tested material for each fraction was about 100 g, and the set time of continuous vibrating was 5 min. As many as five repetitions were completed for every tested material. The bulk density of the particles was also investigated, according to [56], with 2 repetitions of the measurement for every tested particle.

For the particleboard preparation, the following fractions were taken: 8–2 mm for the core layer (CL), 1 mm and below for face layers (FL).

### 2.5. Statistical Analysis

Analysis of variance (ANOVA) and *t*-test calculations were used to test (α = 0.05) for significant differences between factors and levels, using IBM SPSS statistic base (IBM, SPSS 20, Armonk, NY, USA) where appropriate. A comparison of the means was performed when the ANOVA indicated a significant difference by employing the Duncan test. The statistically significant differences in achieved results whenever the data were evaluated are given in the Results and Discussion section.

## 3. Results and Discussion

### 3.1. Fraction Share and Bulk Density of Particles

**Figure 2:**
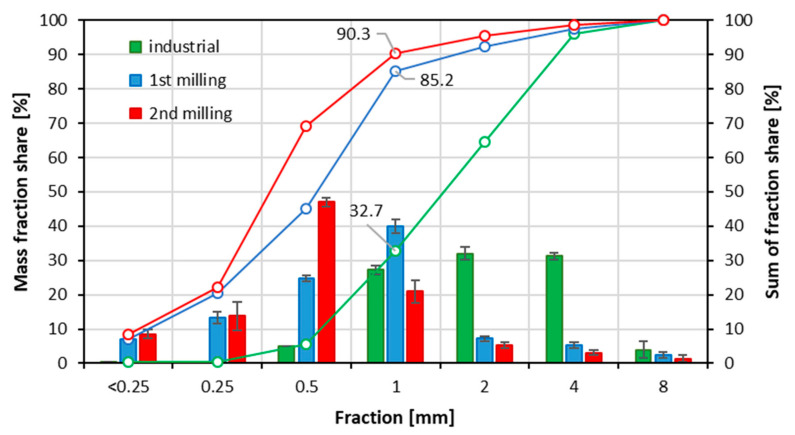
Mass fraction share of the particles used in research.

The fraction share, as well as the sum of the fraction share of the particles used in research, is displayed in Figure 2, and the bulk density is provided in Table 1. The fraction share results show the mass share (referred to as the total mass of the particle sample) of the particles found in the particle mixture after sieving on the meshes of the size indicated on the plot (here as bars). According to the results, in the case of industrial particles, the highest quantity of particles, over 67% is for fractions 2, 4, and 8 mm (see the lines referring to the sum of the fraction share (%)). These fractions are intended to be used for core layer production. Knowing that the core layer weight in 16 mm thick particleboards is about 68% [57], it can be concluded that the achieved fraction share of industrial particles is almost fully proportional to this demanded contribution. When analyzing the fraction share of particles after first milling, a significant increase in fine fraction particle quantity and a decrease in larger dimension particles (2 mm and larger) can be found. The dominating fraction is 1 mm, where 39.9% of particles were found. The sum of <0.25–1 mm fractions of this type of particle, intended to produce the face layers, is 85.2%. Less than 15% of particles are 2 mm or larger. This face-to-core particle mass content ratio is unprofitable in light of the proportions mentioned above of the face and core layer content in particleboards. An worse situation was found in the case of particles of second milling, where 90.3% of particles are fractions smaller than 2 mm, which means only 9.7% of particles are larger than 1 mm and can be used for core layer production. In this case, the highest quantity of particles, 47%, is the fraction of size of 0.5 mm.

The results of bulk density measurement (Table 1) show that the bulk density of the particles significantly increased after first milling. The bulk density increase was 59% for face layer particles and 58% for core layer particles. The further milling (second milling) of the particleboards increased bulk density by about 6% for face layer particles and 11% for core layer particles. The high rise in bulk density of the particles after first milling can be explained by the fact, that the particles have been produced by milling the particleboards of an average density of 680 kg m^−3^, and not by cutting the native *Pinus sylvestris* L. wood of average density of about 485 kg m^−3^ [58], as the industrial particles are produced. This influence of the raw material density on the bulk density of produced particles has been confirmed by [5]. Moreover, the density of the face layers of particleboards is much higher than the average density of the panel and can reach over 800 kg m^−3^ (almost 3000 kg m^−3^ for decorative melamine paper). This can lead to an increase in the bulk density of the particles produced by milling the particleboards; however, the bulk density is much higher after first milling since the second milling is performed on the same particleboard density.

An effect of particle shape change due to the re-milling of particleboards is presented in Table 2, where pictures of the particles are presented. The most important change is visible when comparing the industrial particles and those after first milling. The length-to-width ratio of the industrial particles is significantly higher (for example about 10:1 for 8 mm and about 20:1 for 2 mm) than that of particles after first milling. This can be confirmed for particles of fractions 0.25 mm and larger. It should be pointed out that the particles of fractions smaller than 8 mm represent a high length-to-width ratio (splinter-like) when the shape of the same fractions of particles after first milling is almost square. The further milling (second milling) of the particleboards produced from re-milled particleboards led to reduce the number of splinter-like particles from about 20–30% (see pictures of fractions 1–2 mm in Table 2 after first milling) to almost 0% after second milling. The factor influencing the production of short and square particles after first and second milling is the brittleness of the particleboard, which consists of 10–12% amine, thermoset resin of fragile bonding line. The same finding has been observed by [34] when evaluating the properties of the recycled particles of several alternative raw materials for particleboard production. Ref. [59] confirms the reduction in the size of wood elements after recycling wood-based composites. It is worth adding that the shape and distribution of the particles can significantly influence the particleboard properties [60,61].

### 3.2. Modulus of Rupture and Modulus of Elasticity

**Figure 3:**
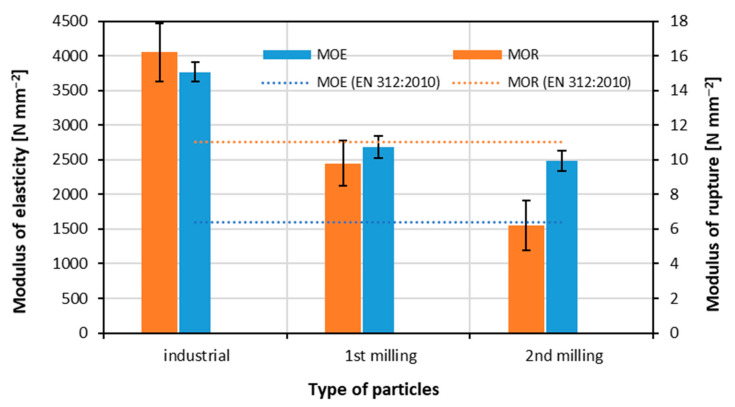
Modulus of elasticity and modulus of rupture of the panels produced by using different particles.

The results of the modulus of rupture and modulus of elasticity measurement are displayed in Figure 3. The MOR values significantly decrease with the subsequent re-milling step, starting from 16.2 N mm^−2^ for industrial particles, through 9.8 N mm^−2^ for panels made of particles after first milling (decrease of about 40%), to 6.2 N mm^−2^ (decrease of about 62% referring to industrial particles panels). When referring to the requirements of [52], only panels made of industrial particles meet the requirements of P2-type panels.

In the case of MOE, a significant reduction was found for panels made of particles taken from first milling (29%), but the further re-milling of the panels to second-milling particles caused the 5% reduction when referring to first-milling particles. This may be caused by the high similarity of first- and second-milling particles, shown in Table 2. All the tested panels meet the minimum MOE requirements of [52] for P2-type panels. Such a high level of MOE, irrespective of multiple re-milled particles, can be caused by the presence of melamine-impregnated decorative paper on the surfaces. This surface finishing material is characterized by high density, thus improving the rigidity of the panel covered by this material. According to [62], the best mechanical properties of wood-based composites can be reached when using particles of a high length-to-thickness ratio. Furthermore, statistically significant differences in the average MOR values were found for all composite types made of different particles. In the case of MOE, statistically significant differences were found between panels made of industrial particles referred to as remaining particles.

### 3.3. Internal Bond and Surface Soundness

**Figure 4:**
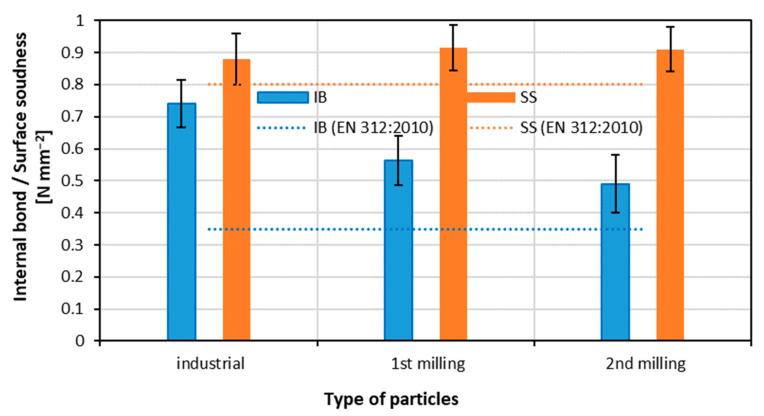
Internal bond and surface soundness of panels produced with different particles.

The results of the measurement of internal bond and surface soundness are presented in Figure 4. In the case of an internal bond, a statistically significant reduction in IB (from 0.74 to 0.56 N mm^−2^) for panels made of first-milling particles was found. The decrease in IB for composites made of second-milling particles was also registered, but the reduction was not statistically significant. The IB decrease in the composites made of re-milled particleboards can be the increase in previously cured resin fine particle content. According to [63], the IB can be enhanced by small particles. The findings of [64] show that an increasing quantity of that fine particles can act as a filler in glue mass. With this type of filler, the previously cured resin particles could not create a unified network structure with the new resin system during the subsequent curing process. According to Rzyska-Pruchnik and Kowaluk [65], the increase in amine resin in particleboards reaches its maximum in the improvement of internal bonds, and a further increase in resination causes a reduction in IB. The decreasing size of the particles can reduce the IB [66]. When analyzing the values of surface soundness, it can be concluded that there is no change in SS when producing panels from re-milled particleboards and referring to the panels made of industrial particles. This stabilization of SS can be the complementary/self-balancing effect of the positive sub-surface layer density increase, which has been found at a thickness of about 0.3 mm, and the negative influence of the fine particles of wood and already cured resin content. It can be concluded that all the achieved results of IB and SS meet the requirements of EN-312 standard [52] for P2-type panels.

### 3.4. Screw Withdrawal Resistance

**Figure 5:**
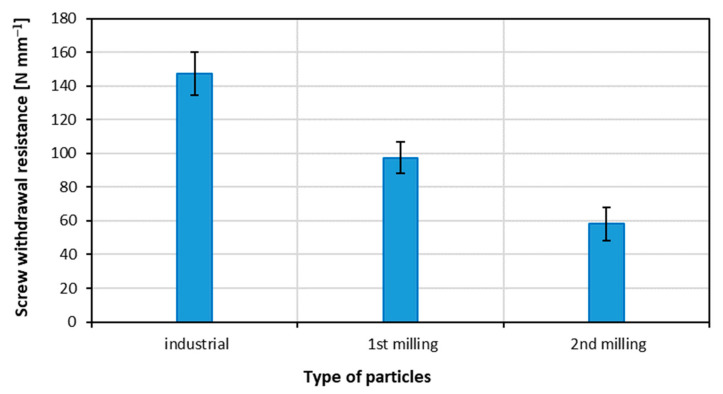
Screw withdrawal resistance of the panels produced using different particles.

The results of the measurement of screw withdrawal resistance are displayed in Figure 5. Statistically significant differences were found for decreased values of SWR with subsequent re-milling of the panels to produce the particleboards out of recovered particles. According to [57,67], the SWR depends mostly on the tested material density and can radically increase with the rise in density. Lubis et al. [68] reported that the screw withdrawal resistance decreases with the increasing quantity of recycled fibers in MDF panels. However, according to [61], the replacement of coarse particles by fine fractions in the core layer of particleboards should provide a rising SWR.

### 3.5. Density Profile

**Figure 6:**
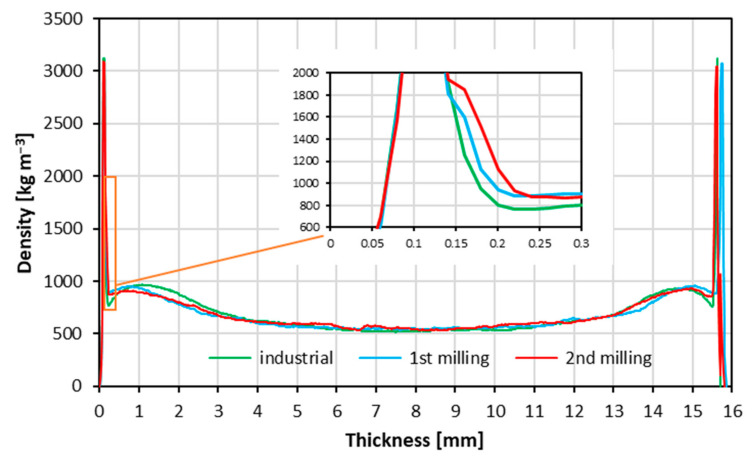
Density profiles of the panels produced using different particles.

The measured density profiles of the tested composites are displayed in Figure 6. The peaks on the face zones (about 3000 kg m^−2^) are the effect of the melamine-impregnated decorative paper presence. The density of the core layers is on the same level for all the tested materials. A slight variation of the density can be found in the face layers, with a thickness of about 0.15–0.30 mm. The results show that the density of the mentioned zone increases with the progressive recycling of the particles from particleboards. This can be the effect of applying fine particles of fine size and the rising bulk density of these particles (according to Table 1). The density of the zone under laminate paper (0.15–0.30 mm) is crucial when analyzing the surface soundness of the composite (Figure 4). This is in line with the findings of [67]. In general, when analyzing the density profiles of wood-based composites, for example, particleboards, it can be concluded that even if the density of the core layer zone is made of raw material of lower bulk density, and the resulting density of that zone is lower, the mechanical parameters of the panel can be high [69]. In the results presented in Figure 6, the density of the core zone of all tested panels is on the same level, so the core zone density should not influence the mechanical features of the panels. However, it should be mentioned that the bulk density of the particles significantly increases after every re-milling (Table 1), which negatively influences the mechanical properties of the panels [34].

### 3.6. Thickness Swelling and Water Absorption

**Figure 7:**
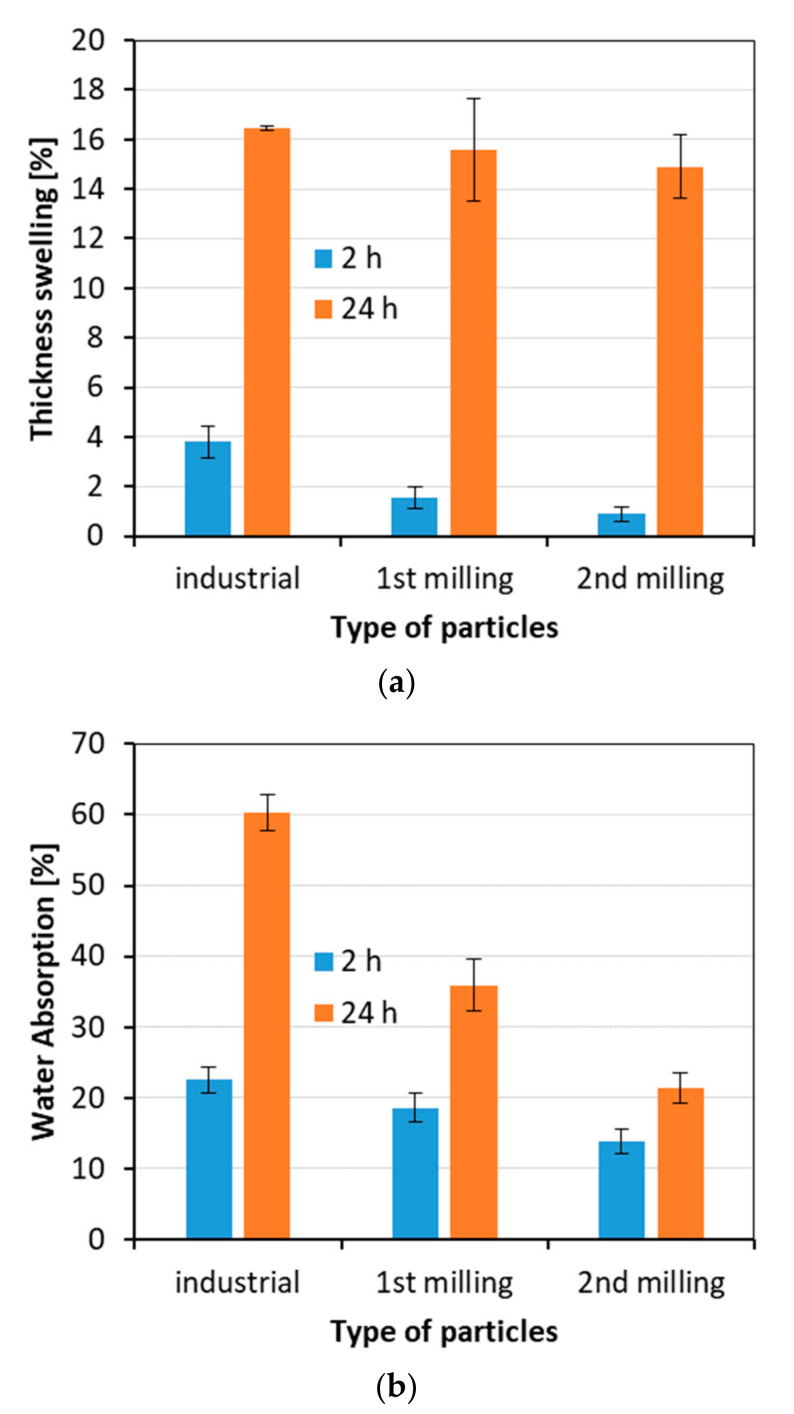
The thickness swelling (**a**) and water absorption (**b**) of the panels produced using different particles.

The results of the measurement of thickness swelling and water absorption are displayed in Figure 7a,b, respectively. The thickness swelling of the tested panels decreases after 2 h, and after 24 h of soaking; however, the decrease after 2 h is more radical than after 24 h. The reason for thickness swelling reduction can be an increasing quantity of resin and hydrophobic agents in the panel structure. It should be mentioned that during the production of every type of tested panels, 1% dry matter of paraffin emulsion was added. Thus, it can be assumed that the content of paraffin increased 3 times when comparing the panels made of industrial particles to panels made of particles after second milling. However, the effect of raising the amount of hydrophobic agent is clearly visible only in the case of 2 h soaking. For longer soaking, the dominant influence is the presence of non-water-resistant resin (UF), which partially reduces the swelling of wood particles but does not prevent this phenomenon. It should be mentioned that in the case of 2 h TS, all the achieved results are significantly different from one another, and in the case of 24 h TS, the only statistically significant difference was found between industrial and second milling panels.

When analyzing the results of water absorption after 2 and 24 h of soaking in water, the reduction in WA after incremental re-milling of the panels to particleboards was found, and this reduction is much more significant than in the case of TS. There are statistically significant differences in mean WA values found between all the tested panels after 2 and 24 h of soaking. The mechanism of water absorption in particleboards is slightly different than that of thickness swelling. Since TS depends mostly on the material density and grows with the density increase, the WA works oppositely. The water can be absorbed mainly by a material of higher porosity where empty spaces are available to collect the water. In regular (non-recycled) wood-based composites, the water absorption is opposite to the internal bond [70]. The decreasing internal bond, which is explained by weaker bond lines between particles, providing more spaces available for water, causes rising water absorption. However, this effect is more complex and can also be influenced by the bulk density of raw material [71]. When referring to the tested panels, it can be concluded that the panels made of particles with an increased number of fine particles lose the ability to collect as much water as the panels made of industrial particles. This is because the fine recycled particles of a higher load of binder previously added to panels fill the zones between the larger particles, which could be empty in the case of regular panels, and thus, the water absorption decreases.

### 3.7. Formaldehyde and TVOC Emission

The results of the measurement of the emission of formaldehyde and TVOC are presented in Table 3. As can be seen, a slight rise in the HCHO and TVOC emissions was found. The reason for the increase in emissions can be the rising content of both already cured resin in re-milled particles as well as freshly added resin. It should be mentioned that in the case of second milled particle composite, the content of the resin is 3 times higher than that of panels made of industrial particles. However, the increase in emissions is not as high as anticipated due to the fact that the panels’ surfaces are covered by melamine-impregnated decorative paper, which acts partially as a barrier layer avoiding increased emission. Since the narrow sides of the tested panels were sealed during the test procedure, the wide surfaces covered by decorative paper are the only zones of formaldehyde and TVOC emission. According to [72], the increased addition of melamine-impregnated paper to raw materials when producing particleboards leads to a significant increase in formaldehyde content. This increased tendency has been confirmed by [73] when analyzing the properties of the panels made using recycled MDF fibers. The slightly increasing formaldehyde emission can be the effect of partial hydrolysis of the cured resin particles [74], which may happen, for example, during the pressing of the mat created of the re-milled particles blended with freshly added UF resin-based binder, containing over 34% water. It should be pointed out that the UF resin used in this research has the lowest stability against hydrolysis.

## 4. Conclusions

The novelty of this research is the approach to recycling the raw materials from particleboards in fully controlled conditions, providing the characterization of produced particles and producing the particleboards with close-to-industrial parameters, and, finally, evaluating the features of produced particleboards in the light of raw materials used.

The results show a significant decrease in the size/dimensions of produced particles after the re-milling of particleboards, which leads to the unprofitable production of fine fractions. The shape of produced particles changes in the direction of shortening. The progressive milling of the particleboards leads to achieving a fraction of the fine-size particles of growing bulk density, which influences the density profile of the panels produced, especially of the face zone. This local densification allows the surface soundness to be kept high, irrespective of the decrease in other mechanical parameters, such as internal bond and screw withdrawal resistance. The bending properties decrease; however, the modulus of rupture decrease is more significant than the modulus of elasticity. The water absorption significantly decreases with the subsequent milling of the panels to produce particleboards. There is also a reduction in thickness swelling, which is most visible in the case of 2 h soaking. There is an insignificant increase in the emission of formaldehyde and TVOC for panels made of re-milled particles, which can be caused by the increasing content of UF resin and partial hydrolysis of previously cured binder.

The results confirmed that subsequent mechanical recycling of particleboards, where the further panels are made fully of second-milling particles, leads to an unprofitable and unacceptable reduction in the mechanical properties of the panels. The physical parameters, such as thickness swelling and water absorption, are improved, but this can be the result of increased content of chemical ingredients, which negatively influence the hygienic features of panels (emission of formaldehyde and TVOC). Nevertheless, when trying to find the optimal application of the panels made of multi re-milled panels, the means of use without special demand regarding high mechanical properties can be suggested. Thus, as an example, these kinds of panels can be applied as a filling material in doors construction, providing acceptable features of mechanical strength and thermal and acoustic insulation. Other applications for non-structural use can be considered for these panels. Further research should be directed towards estimating the optimal addition ratio of mechanically recycled particles to particleboard production. The identification and characterization methods of raw materials should be developed to characterize the raw materials used in particleboard plants.

## Figures and Tables

**Table 1:** The average values and standard deviation of selected research results.

Type of Particles	MOR	MOE	IB	SS	SWR	TS	WA	Bulk Density
	N mm^−2^				N mm^−1^	%		kg m^−3^
Industrial	16.2 (1.7) *	3767 (136)	0.74 (0.07)	0.88 (0.08)	147 (13)	2 h: 3.8 (0.7) 24 h: 16.4 (0.1)	2 h: 22.6 (1.8) 24 h: 60.2 (2.5)	FL **: 175 CL: 150
First milling	9.8 (1.3)	2679 (157)	0.56 (0.08)	0.92 (0.07)	97 (9)	2 h: 1.6 (0.4) 24 h: 15.6 (2.1)	2 h: 18.6 (2.1) 24 h: 35.9 (3.7)	FL: 278 CL: 237
Second milling	6.2 (1.4)	2483 (142)	0.49 (0.09)	0.91 (0.07)	58 (10)	2 h: 0.9 (0.3) 24 h: 14.9 (1.3)	2 h: 13.8 (1.7) 24 h: 21.4 (2.1)	FL: 289 CL: 254
P2 [52]	11	1600	0.35	0.8	n/a	n/a	n/a	n/a

* The data in brackets are standard deviation values; ** FL—face layers, CL—core layers; n/a—not applicable.

**Table 2:** The pictures of the particles used in research (dimensions of pictures: 100 × 100 mm^2^).

Fraction (mm)	Type of Particles		
	Industrial	First Milling	Second Milling
8	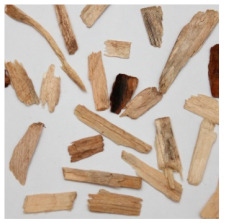	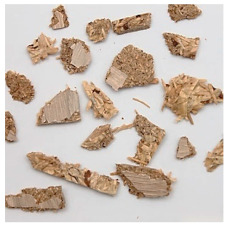	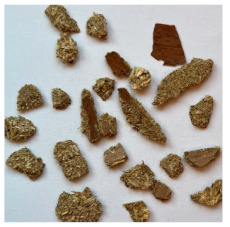
4	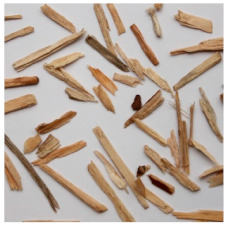	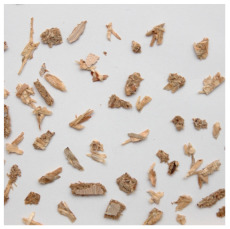	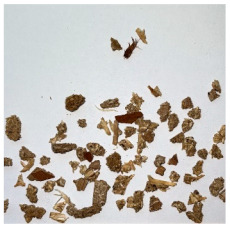
2	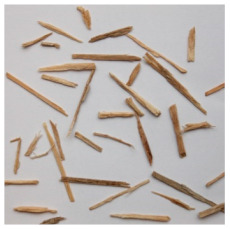	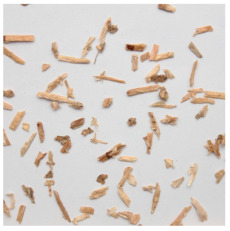	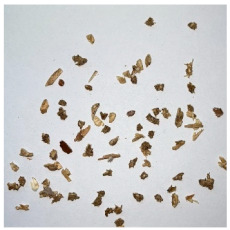
1	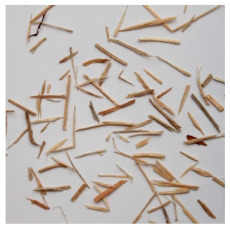	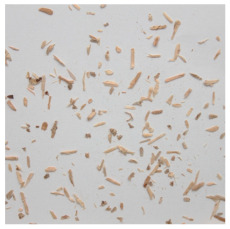	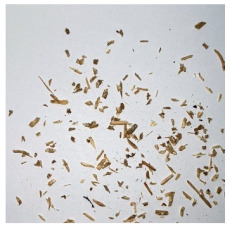
0.5	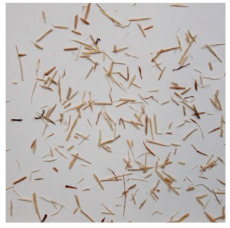	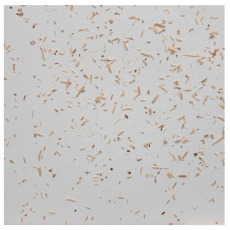	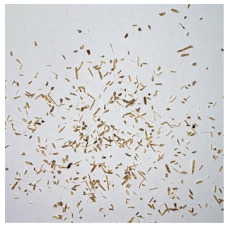
0.25	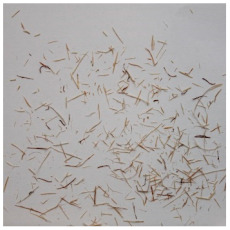	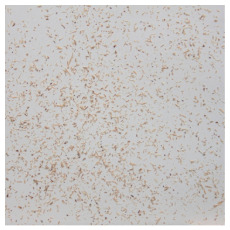	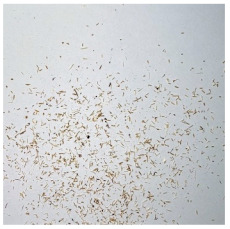
<0.25	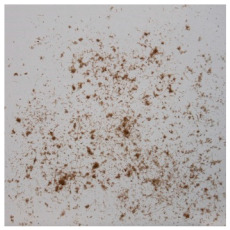	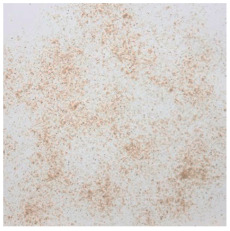	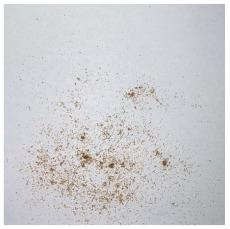

**Table 3:** The emission of HCHO and TVOC of the tested panels produced using different particles.

Type of Particles	HCHO	TVOC
	mg m^−3^	
Industrial	0.072	0.084
First milling	0.081	0.086
Second milling	0.087	0.092

## Data Availability

The data presented in this study are available on request from the corresponding author.
